# Prevalence of Diabetes, Ketosis, and Ketoacidosis and Their Correlation With Mortality in Critical COVID-19 Patients: A Single-Center Retrospective Study

**DOI:** 10.7759/cureus.57551

**Published:** 2024-04-03

**Authors:** Mohamed F Hendi, Zeyad F Alrais, Fahimuddin Syed, Hesham M Elkholy, Hawra Alsayed, Muneeba Moin, Sara H Mukhtar

**Affiliations:** 1 Intensive Care Unit, Rashid Hospital, Dubai, ARE

**Keywords:** icu, intensive care unit, ketoacidosis, covid-19, ketosis, diabetes

## Abstract

Aim

We aimed to find out the prevalence of diabetes, ketosis, and ketoacidosis in coronavirus disease 2019 (COVID-19) critically ill patients and to explore the clinical impact of the development of ketosis and ketoacidosis on the outcome of COVID-19 critically ill patients and identify them as potential risk factors for these patients.

Methods

We collected data on COVID-19 patients admitted to the intensive care unit (ICU) retrospectively. The study population will be classified into two groups based on the presence of diabetes or ketosis.

Results

The study comprises data on 253 ICU patients admitted with COVID-19 pneumonia. Two hundred patients (79.05%) had diabetes or prediabetes on admission. Seventy-six patients (30%) presented with ketosis. Nine patients had progressed to diabetic ketoacidosis during their ICU stay. Concerning the outcome, among 150 patients who died (59.3%), there was significantly higher mortality among the ketotic patients (69.7%) compared to nonketotic patients (54.8%) with a P-value < 0.027. We noted that the peak blood glucose level during ICU stay was statistically significantly higher in nonsurvivors (mean 345 mg/dl) compared to survivors (mean 298 mg/dl) with a P-value of 0.006. Our data showed that peak serum levels of lactate, procalcitonin (PCT), C-reactive protein, white blood cells (WBC), D dimer, and lactate dehydrogenase strongly positively correlated to the length of ICU stay. We used the ROC curve (receiver operating characteristic curve) to assess the relation between many laboratories and mortality. We noted that uncontrolled hyperglycemia and other laboratory variables are significant predictors of mortality of COVID-19 patients (e.g., peak blood glucose (P = 0.004), PCT (P = 0.047), and P < 0.001 of other laboratories (e.g. lactate, PH, WBC, D dimer, ferritin).

Conclusion

We reported a high prevalence of diabetes and ketosis among COVID-19 patients admitted to the ICU. Ketosis is associated with an increased mortality risk. Uncontrolled hyperglycemia is a significant predictor of mortality in critically ill COVID-19 patients.

## Introduction

During the coronavirus disease 2019 (COVID-19) outbreak in 2020, there was a huge increase in demand for critical ill services owing to the massive growth in the number of patients with severe illness during the pandemic.

Diabetes is a common comorbidity in COVID-19 patients due to its high prevalence worldwide and occurs in around 20-50% of patients, depending on the global region [1]. We wanted to review and analyze evidence on the relationship between COVID-19 and diabetes patients.

There is limited data on ketosis and ketoacidosis in critically ill COVID-19 patients. We report many cases of ketosis and diabetic ketoacidosis (DKA) precipitated by COVID-19 infection. Some COVID-19 patients present with ketosis, which may progress to DKA in those with diabetes.

Diabetes is a chronic inflammatory disorder marked by a variety of metabolic and vascular problems that can impair the ability to respond to infections. Serious acute metabolic complications, such as DKA, are often caused by infections and linked to uncontrolled blood glucose.

Previous viral pandemics have shown that diabetes is associated with higher rates of mortality and morbidity. Diabetes was identified as a major contributor to disease severity and mortality in the 2012 outbreak of Middle East Respiratory Syndrome Coronavirus (MERS-CoV) [1]. Similarly, the presence of diabetes tripled the risk of hospitalization and quadrupled the risk of intensive care unit (ICU) admission during Inﬂuenza A (H1N1) infection outbreak in 2009 [2]. Diabetes and uncontrolled glycemia have been reported as significant predictors of severity and deaths in patients infected with different previous pandemic viruses, including the pandemic influenza A (H1N1), MERS-CoV, and SARS-CoV-1 [3].

This study will focus on the prevalence of diabetes, ketosis, and ketoacidosis in critical adult patients with COVID-19 pneumonia who were admitted to the ICU and their correlation with ICU mortality to provide a better understanding of COVID-19 in diabetic patients.

## Materials and methods

Population and study sample

We enrolled all adult critically ill patients who were diagnosed with COVID-19 pneumonia and admitted to the ICU of our hospital (Rashid Hospital) in Dubai during the COVID-19 pandemic from January 1, 2020, to June 30, 2021, in our study, which comprised 253 critically ill patients. During the pandemic, oral and nasal swabs were collected for SARS-CoV-2 PCR for any suspected cases of COVID-19 pneumonia.

We collected epidemiological and clinical data as well as data from laboratory investigations and outcomes from the Salama Electronic medical record system. We collected results of laboratories done on the day of ICU admission e.g. blood glucose, lactate, PH, PCT, CRP, WBC, D dimer, ferritin, and lactate dehydrogenase (LDH) and also results of the highest peak level of those laboratories during the whole ICU stay via retrospective collection of laboratories flowchart of our electronic hospital file system. We analyzed retrospectively collected data using Microsoft Excel 2016.

We classified the study population into two groups based on the presence of diabetes or ketosis. Ketosis is defined by a positive test result of urine or serum ketone (>0.6 mmol/l), whereas ketoacidosis is defined by a positive test result of urine or serum ketone (>0.6 mmol/l) with arterial pH < 7.35 and/or bicarbonate level < 18 mmol/L [4]. We statistically analyzed and expressed data as medians, percentages, and P-values to assess the differences between groups, such as ketosis against nonketosis and diabetes versus nondiabetes.

Statistical methods

We coded and entered data using IBM SPSS Statistics for Windows, Version 28 (Released 2021; IBM Corp., Armonk, New York, United States). For categorical data, we used frequency (count) and relative frequency (%) to summarize the data, whereas for quantitative data, we used mean, standard deviation, median, minimum, and maximum.

We used the Mann-Whitney and nonparametric Kruskal-Wallis tests to compare quantitative variables [5]. To compare categorical data, we used the Chi-square (c2) test. When the expected frequency was less than 5, we used the exact test instead [6].

We used the Spearman correlation coefficient [7] to determine correlations between quantitative variables. Spearman correlation coefficient (rho) (r) shows the strength and direction of the correlation. If rho is positive, then the direction of the correlation is positive, and if it is negative, then the direction of the correlation will be negative.

The strength of the correlation is determined by the amount of rho, irrespective of its sign. If the rho value < 0.3, there is a weak correlation. If the rho value is 0.3-0.49, there is a moderate correlation. If the rho value < 0.5, there is a strong correlation.

The receiver operating characteristic (ROC) curve will be calculated to assess the relation between many laboratories e.g. Blood glucose, lactate, PH, PCT, CRP, WBC, D dimer, ferritin, LDH, and mortality. To detect the best cutoff value for the detection of the outcome of significant parameters, we constructed the ROC curve and performed an analysis with the area under the curve (AUC).

P-values less than 0.05 were considered statistically significant. P-values less than 0.001 were considered highly statistically significant.

## Results

The study was conducted on a total of 253 patients admitted to the ICU with COVID-19 pneumonia. The study population was classified into categories (diabetic or prediabetic versus nondiabetic and ketosis versus nonketosis) as shown in Table 1. The prevalence of complications associated with COVID-19 in our study is shown in Table 1.

**Table 1:** Sex, group distribution, and complications associated with COVID-19 infection in our study ARDS: Acute respiratory distress syndrome; AKI: acute kidney injury; MI: myocardial infarction; ICH: intracerebral hemorrhage; COVID-19: coronavirus disease 2019

Study Group Distribution		Count	%
Sex	M	198	78.3%
	F	55	21.7%
Diabetic/prediabetic	yes	153	60.5%
	no	100	39.5%
Ketosis/ketonuria	yes	76	30.0%
	no	177	70.0%
Diabetic ketoacidosis	yes	9	3.6%
	no	244	96.4%
Other complications		Count	%
ARDS	yes	201	79.4%
	no	52	20.6%
Septic shock	yes	40	15.8%
	no	213	84.2%
AKI	yes	95	37.5%
	no	158	62.5%
Pneumothorax	yes	19	7.5%
	no	234	92.5%
MI	yes	8	3.2%
	no	245	96.8%
Thrombotic disease	yes	21	8.3%
	no	232	91.7%
Bleeding	yes	16	6.3%
	no	237	93.7%
ICH	yes	7	2.8%
	no	246	97.2%

Our patients had a median age of 53 (22-92) years; 198 patients were male (78.3%). Two hundred (79.05%) ICU patients had diabetes or prediabetic on admission, and they had a median age of 54 (24-92) years. Patients with diabetes were older (median age 54 vs. 46 years; P-value < 0.001).

In this study, seventy-six (30%) patients presented with ketosis. They had a median age of 51 (24-78) years. Nine of the ketosis group had progressed to diabetic ketoacidosis during ICU admission. Eight patients with diabetic ketoacidosis died.

We observed that there was no statistical difference in the duration of ICU stay between the diabetic and nondiabetic groups (median 15 (1-197) vs. 16 (1-327) days; P = 0.985), as shown in Table 2, and also between the ketosis and nonketosis groups (median 16 (1-115) vs. 14 (1-327) days; P = 0.335), as shown in Table 3.

**Table 2:** Age, duration of ICU stay, and laboratory results of patients with or without diabetes who were infected with COVID-19 COVID-19: Coronavirus disease 2019; ICU: intensive care unit; CRP: C-reactive protein; LDH: lactate dehydrogenase

Variables	Diabetes										
	Yes					No					P-value
	Mean	SD	Median	Minimum	Maximum	Mean	SD	Median	Minimum	Maximum	
Age	55.27	12.76	54.00	24.00	92.00	48.25	15.24	46.00	22.00	89.00	< 0.001
Length of ICU stay	25.22	33.59	15.00	1.00	197.00	24.54	39.14	16.00	1.00	327.00	0.985
HBA1C	8.54	2.47	7.70	5.30	18.30	6.73	2.22	6.10	4.60	17.70	< 0.001
Ketonuria	0.59	0.97	0.00	0.00	4.00	0.32	0.80	0.00	0.00	4.00	0.010
Glycosuria	1.93	1.68	2.00	0.00	4.00	0.66	1.30	0.00	0.00	4.00	< 0.001
Blood glucose on admission	231.73	110.49	203.00	52.00	600.00	176.81	117.97	152.00	65.00	1122.00	< 0.001
Peak blood glucose	362.63	101.73	344.00	112.00	600.00	270.19	123.94	248.00	99.00	1122.00	< 0.001
PH on admission	7.37	0.14	7.42	6.73	7.53	7.35	0.13	7.38	6.96	7.55	0.100
PH peak	7.15	0.15	7.18	6.69	7.41	7.12	0.18	7.14	6.71	7.48	0.196
Lactate on admission	2.60	3.09	1.80	0.60	26.00	2.58	2.42	1.80	0.60	16.00	0.536
Lactate peak	7.64	5.99	4.60	1.10	27.00	7.83	5.72	5.60	2.20	25.00	0.448
Procalcitonin on admission	5.40	26.34	0.39	0.04	269.56	1.72	4.82	0.41	0.03	33.19	0.480
Procalcitonin peak	16.48	33.66	3.48	0.10	269.56	28.33	66.25	4.66	0.21	352.00	0.301
CRP on admission	155.58	115.23	131.00	0.50	497.90	156.87	109.41	145.60	1.70	514.00	0.736
CRP peak	271.52	143.06	247.60	18.10	727.00	267.96	130.18	280.00	3.30	563.00	0.706
WBC on admission	9.72	4.84	8.20	2.50	26.40	11.49	6.05	10.40	2.80	39.00	0.006
WBC peak	25.10	10.96	23.90	6.90	60.30	27.31	12.44	23.40	7.70	69.70	0.241
Ferritin on admission	1602.77	3781.14	954.85	18.00	43678.00	1996.76	3077.49	1308.00	25.00	21890.00	0.015
Ferritin peak	6105.17	19244.35	1748.15	72.00	151800.00	4595.79	5765.40	2567.00	231.20	29891.00	0.002
D dimer on admission	4.17	5.86	1.51	0.20	20.00	4.60	5.87	1.98	0.24	20.00	0.123
D dimer peak	9.98	7.29	7.22	0.68	20.00	11.31	7.31	10.48	1.08	20.00	0.155
LDH on admission	453.45	213.43	399.00	173.00	1907.00	587.91	579.16	515.00	94.00	5732.00	0.003
LDH peak	720.71	982.55	605.00	173.00	11864.00	954.02	1424.50	687.00	141.00	12512.00	0.004

**Table 3:** Age, duration of ICU stays, and laboratory results of patients with ketosis or nonketotic who were infected with COVID-19 ICU: Intensive care unit; COVID-19: coronavirus disease 2019; CRP: C-reactive protein; LDH: lactate dehydrogenase

Variables	Ketosis/ketonuria										
	Yes					No					P-value
	Mean	SD	Median	Minimum	Maximum	Mean	SD	Median	Minimum	Maximum	
Age	52.49	11.72	51.00	24.00	78.00	54.36	14.31	53.00	22.00	92.00	0.488
Length of ICU stay	23.26	23.80	16.00	1.00	115.00	25.67	39.92	14.00	1.00	327.00	0.335
HBA1C	8.98	2.97	8.45	4.60	18.30	7.54	2.16	6.80	4.80	15.70	< 0.001
Ketonuria	1.53	1.04	1.00	0.00	4.00	0.00	0.00	0.00	0.00	0.00	< 0.001
Glycosuria	2.36	1.74	3.00	0.00	4.00	1.01	1.44	0.00	0.00	4.00	< 0.001
Blood glucose on admission	257.13	161.06	220.00	55.00	1122.00	189.79	83.56	169.00	52.00	473.00	0.001
Peak blood glucose	385.30	143.46	368.00	99.00	1122.00	300.67	97.88	290.00	112.00	600.00	< 0.001
PH on admission	7.36	0.12	7.40	6.77	7.52	7.37	0.14	7.41	6.73	7.55	0.433
PH peak	7.14	0.16	7.18	6.74	7.37	7.13	0.17	7.16	6.69	7.48	0.664
Lactate on admission	2.27	2.22	1.70	0.90	17.00	2.73	3.06	1.80	0.60	26.00	0.321
Lactate peak	7.37	5.63	4.60	2.00	25.00	7.86	5.99	5.40	1.10	27.00	0.632
Procalcitonin on admission	7.72	33.21	0.45	0.06	269.56	2.33	11.76	0.37	0.03	120.84	0.090
Procalcitonin peak	22.17	41.61	4.31	0.26	269.56	20.69	52.41	3.58	0.10	352.00	0.114
CRP on admission	159.17	117.31	118.20	0.70	433.70	154.77	111.05	140.60	0.50	514.00	0.944
CRP peak	281.80	143.87	278.10	18.10	587.70	265.08	135.33	266.40	3.30	727.00	0.340
WBC on admission	10.81	5.09	9.80	2.50	26.40	10.25	5.54	8.60	2.80	39.00	0.182
WBC peak	26.42	11.07	24.30	8.90	69.70	25.78	11.84	23.40	6.90	65.90	0.448
Ferritin on admission	1870.80	2420.57	1385.65	55.60	15588.00	1711.94	3899.67	1040.45	18.00	43678.00	0.070
Ferritin peak	5743.89	9262.99	2310.00	72.00	54200.00	5400.61	17359.22	1957.50	72.70	151800.00	0.126
D Dimer on admission	4.60	5.86	1.86	0.20	20.00	4.22	5.87	1.70	0.24	20.00	0.460
D Dimer peak	11.28	7.24	12.19	1.29	20.00	10.17	7.33	8.10	0.68	20.00	0.321
LDH on admission	471.91	181.65	444.00	202.00	1294.00	523.09	472.96	443.00	94.00	5732.00	0.922
LDH peak	704.45	400.57	647.50	294.00	3258.00	862.19	1392.61	635.50	141.00	12512.00	0.821

**Figure 1:**
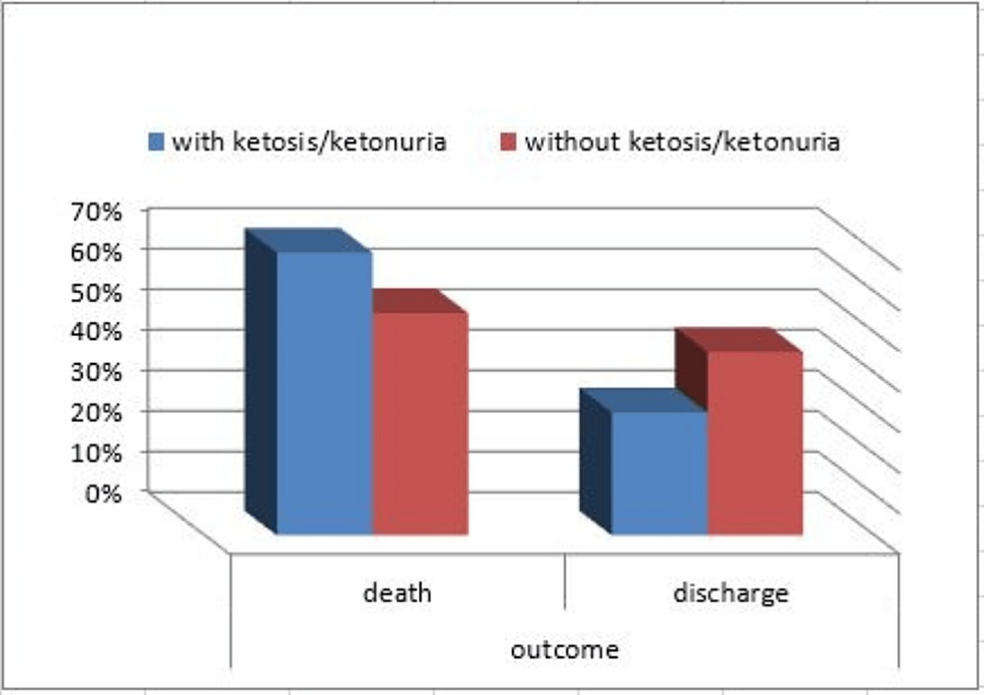
Mortality and discharge distribution among studied groups (ketosis and nonketosis)

**Figure 2:**
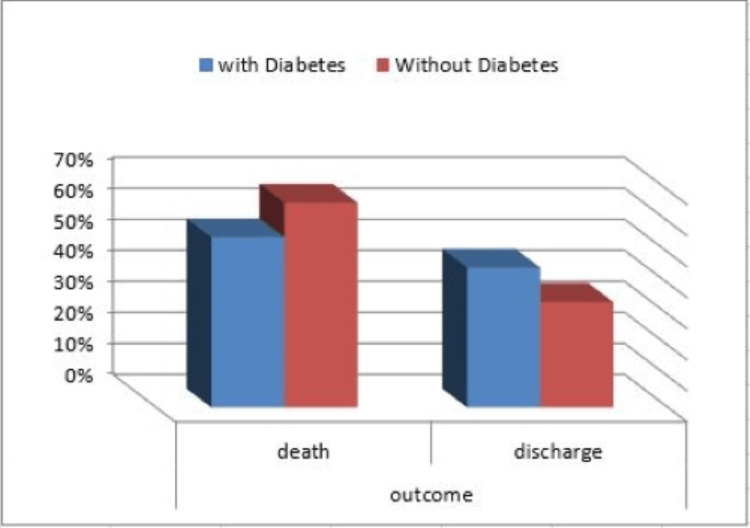
Mortality and discharge distribution among studied groups (diabetic and nondiabetic)

Concerning the outcome of the 253 COVID-19 patients included in our study, 150 patients died (59.3%), and 103 patients were discharged from ICU (40.7%). According to the mortality outcome, patients were divided into survivors and nonsurvivors. Our data showed there was a significantly higher mortality among the ketotic patients (69.7%) compared to nonketotic patients (54.8%) with a P-value < 0.027, as shown in Table 4 and Figure 1. However, there was no significant mortality difference among the diabetic group (54.9%) compared to the nondiabetic group (66%) (Figure 2).

**Table 4:** Mortality and discharge distribution among studied groups

Group		Outcome				
		Death		Discharge		P-value
		Count	%	Count	%	
Diabetic/prediabetic	yes	84	54.9%	69	45.1%	0.079
	no	66	66.0%	34	34.0%	
Ketosis/ketonuria	yes	53	69.7%	23	30.3%	0.027
	no	97	54.8%	80	45.2%	

We noted that peak blood glucose level was statistically significantly higher in nonsurvivors (mean 345 mg/dl) compared to survivors (mean 298 mg/dl) with a P-value of 0.006, while HBA1C and blood glucose on admission showed no statistical difference in ICU outcomes, as shown in Table 5.

**Table 5:** Distribution of ketonuria, glycosuria, and laboratory results among survivors and nonsurvivors CRP: C-reactive protein; LDH: lactate dehydrogenase

Laboratories	Outcome										
	Death (nonsurvivors)					Discharge (survivors)					P-value
	Mean	SD	Median	Minimum	Maximum	Mean	SD	Median	Minimum	Maximum	
HBA1C	8.28	2.73	7.30	4.60	18.30	7.63	2.20	6.70	4.80	14.30	0.090
Ketonuria	0.56	1.01	0.00	0.00	4.00	0.37	0.76	0.00	0.00	3.00	0.127
Glycosuria	1.54	1.68	1.00	0.00	4.00	1.28	1.63	0.00	0.00	4.00	0.121
Blood glucose on admission	223.72	134.21	185.50	52.00	1122.00	190.07	80.59	167.00	65.00	406.00	0.074
Peak blood glucose	345.33	129.66	328.00	137.00	1122.00	298.08	97.47	294.00	99.00	600.00	0.006
PH on admission	7.35	0.13	7.38	6.77	7.55	7.38	0.14	7.42	6.73	7.53	0.021
PH peak	7.09	0.16	7.12	6.71	7.35	7.20	0.14	7.24	6.69	7.48	< 0.001
Lactate on admission	2.61	2.15	1.90	0.60	13.10	2.57	3.63	1.60	0.60	26.00	0.009
Lactate peak	9.82	6.20	8.80	1.10	27.00	4.65	3.60	3.40	2.20	26.00	< 0.001
Procalcitonin on admission	3.89	23.45	0.42	0.04	269.56	4.03	16.17	0.37	0.03	120.84	0.125
Procalcitonin peak	26.62	60.02	5.68	0.10	352.00	13.19	25.56	2.88	0.11	139.08	0.043
CRP on admission	161.49	108.69	140.80	0.60	426.60	148.23	118.51	126.40	0.50	514.00	0.215
CRP peak	272.44	149.15	277.00	3.30	727.00	266.77	120.37	247.60	26.80	661.60	0.851
WBC on admission	10.79	5.89	9.75	2.70	39.00	9.87	4.59	8.30	2.50	22.00	0.293
WBC peak	27.99	12.42	26.40	6.90	69.70	23.05	9.61	21.20	7.70	65.90	< 0.001
Ferritin on admission	2087.79	4130.03	1288.00	18.00	43678.00	1280.76	2286.46	954.85	20.80	21890.00	0.001
Ferritin peak	7838.26	19518.34	2626.50	72.00	151800.00	2095.68	2466.11	1681.50	94.00	21890.00	< 0.001
D dimer on admission	5.02	6.30	1.98	0.40	20.00	3.37	5.05	1.50	0.20	20.00	0.031
D dimer peak	11.87	7.11	12.53	0.68	20.00	8.59	7.18	4.95	0.98	20.00	< 0.001
LDH on admission	511.25	225.45	490.00	141.00	1572.00	501.59	579.22	406.50	94.00	5732.00	0.013
LDH peak	898.25	1454.87	648.00	141.00	12512.00	691.00	577.72	632.00	241.00	5732.00	0.165

**Figure 3:**
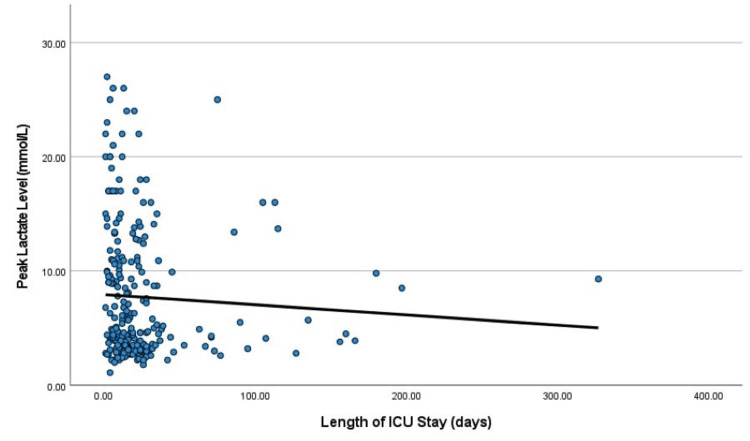
Correlation between the peak lactate level and length of ICU stay

Our data showed that peak serum levels of lactate, PCT, CRP, WBC, D dimer, and LDH during ICU stay strongly positively correlated to the length of ICU stay (Figure 3). However, there was no statistically significant positive correlation between laboratory results on admission e.g. blood glucose, lactate, PH, PCT, CRP, WBC, D dimer, ferritin, LDH, and the length of ICU stay, as shown in Table 6.

**Table 6:** Correlation between ketonuria and glycosuria, and other laboratory results and length of ICU stay

Laboratories	Length of ICU Stay		
	Correlation Coefficient	P-value	N
HBA1C	-0.004	0.956	214
Ketonuria	-0.004	0.956	242
Glycosuria	-0.033	0.612	242
Blood glucose on admission	-0.013	0.838	253
Peak blood glucose	0.035	0.582	253
PH on admission	0.118	0.060	253
PH peak	-0.040	0.529	253
Lactate on admission	-0.098	0.121	253
Lactate peak	-0.154	0.014	253
Procalcitonin on admission	-0.027	0.666	253
Procalcitonin peak	0.209	0.001	252
CRP on admission	0.049	0.438	253
CRP peak	0.315	< 0.001	252
WBC on admission	-0.088	0.162	253
WBC peak	0.263	< 0.001	252
Ferritin on admission	-0.028	0.657	246
Ferritin peak	0.086	0.177	246
D dimer on admission	-0.038	0.556	248
D dimer peak	0.137	0.031	248
LDH on admission	-0.001	0.993	247
LDH peak	0.135	0.033	248

**Figure 4:**
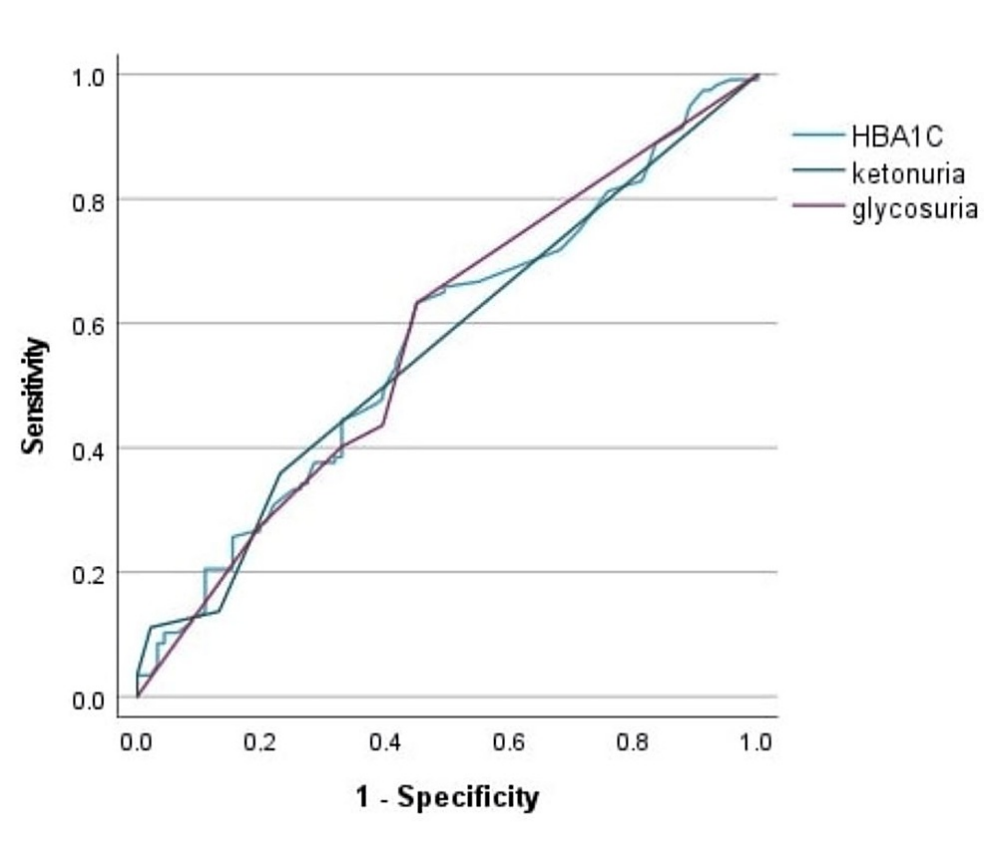
ROC curve for HBA1C, ketonuria, and glycosuria on admission as a prognostic marker in COVID-19 infections to predict ICU mortality ROC: Receiver operating characteristic; COVID-19: coronavirus disease 2019; ICU: intensive care unit

The ketosis group had higher mortality during COVID-19 infection than nonketosis groups, as shown in Table 4. For more optimum results, we calculated the ROC curve to assess the relation between glycosuria, ketonuria, and HBA1C levels and mortality. We observed that there is no significant P-value to predict ICU mortality, as shown in Table 7 and Figure 4.

**Table 7:** Prognostic ability of HBA1C, ketonuria, and glycosuria in COVID-19 infection COVID-19: Coronavirus disease 2019

Mortality	Area under the curve	P-value	95% Confidence interval	
			Lower bound	Upper bound
HBA1C	0.563	0.118	0.484	0.641
Ketonuria	0.560	0.136	0.481	0.638
Glycosuria	0.572	0.075	0.493	0.651

**Figure 5:**
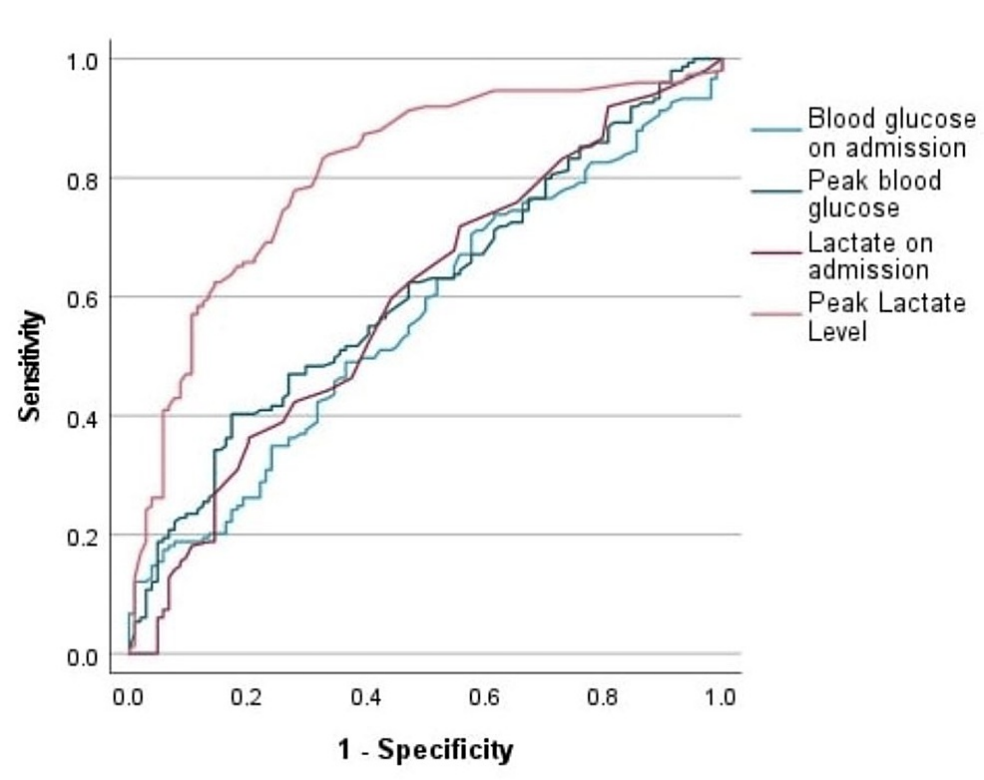
ROC curve for PH, lactate, and blood glucose (peak, on admission) as a prognostic marker in COVID-19 infections to predict ICU mortality ROC: Receiver operating characteristic; COVID-19: coronavirus disease 2019; ICU: intensive care unit

We calculated the ROC curve to assess the relationship between laboratory results and mortality. We noted that uncontrolled hyperglycemia during ICU admission (peak blood glucose) and other laboratory variables are significant predictors of mortality during ICU admission for patients with COVID-19, as shown in Table 8 and Figure 5).

**Table 8:** Prognostic ability of laboratory results (peak, on admission) in COVID-19 infections CRP: C-reactive protein; COVID-19: coronavirus disease 2019; LDH: lactate dehydrogenase

Mortality	Area under the curve	P-value	95% Confidence interval		Cutoff value	Sensitivity %	Specificity %	PPV %	NPV %	Accuracy %
			Lower bound	Upper bound						
Lactate on admission	0.597	0.008	0.526	0.668	1.75	60	56.3	66.67	49.15	58.50
Lactate peak	0.805	< 0.001	0.749	0.860	4.05	82.7	67	78.48	72.63	76.28
Blood glucose on admission	0.566	0.068	0.495	0.637	----	-----	-----	----	----	-----
Peak blood glucose	0.602	0.004	0.532	0.672	322	51.3	63.1	66.96	47.10	56.13
PH on admission	0.585	0.018	0.514	0.656	7.4020	56.7	60.2	67.46	48.82	58.10
PH peak	0.721	< 0.001	0.657	0.786	7.2105	78.7	62.1	75.16	66.67	71.94
Procalcitonin on admission	0.557	0.131	0.483	0.630	----	-----	-----	----	----	-----
CRP on admission	0.546	0.218	0.473	0.619	----	-----	-----	----	----	-----
WBC on admission	0.539	0.289	0.467	0.611	----	-----	-----	----	----	-----
Procalcitonin peak	0.573	0.047	0.501	0.644	4.485	54.1	61.2	66.94	47.73	56.92
CRP peak	0.505	0.893	0.433	0.576	----	-----	-----	----	----	-----
WBC peak	0.635	< 0.001	0.566	0.704	24.05	60.1	67	72.58	53.49	62.85
Ferritin on admission	0.624	0.001	0.552	0.695	1297.5	50.7	65.7	68.47	47.89	56.92
D dimer on admission	0.588	0.018	0.515	0.661	1.61	59.9	57.6	67.16	49.58	58.89
LDH	0.592	0.015	0.518	0.666	456.5	54.9	60.6	66.67	47.69	56.92
Ferritin peak	0.691	< 0.001	0.626	0.757	2598.5	52.1	79	78.00	52.94	62.85
D dimer peak	0.635	< 0.001	0.564	0.707	7.935	64.8	62	71.32	54.70	63.64
LDH peak	0.556	0.131	0.483	0.629	----	-----	-----	----	----	-----

## Discussion

Diabetes is a chronic inflammatory condition characterized by multiple metabolic and vascular abnormalities that can affect the human response to pathogens [8]. Diabetes is one of the most important comorbidities associated with the severity of all three known human pathogenic coronavirus infections, including the 2009 pandemic influenza A (H1N1), MERS-CoV, and SARS-CoV [1]. During the 2012 outbreak of MERS-CoV, diabetes was prevalent in nearly 50% of the population [9]. Diabetes is a common comorbidity in SARS-CoV-2 (COVID-19) patients due to the high frequency of the disease worldwide [10]. Depending on the worldwide region, 20-50% of patients in the COVID-19 pandemic had diabetes [1].

We carried out this study on 253 critically ill patients admitted to ICU with COVID-19 pneumonia. We classified the study population into two groups based on the presence of diabetes or ketosis. Our study aimed to provide a better understanding of COVID-19 in diabetic patients and to focus on issues related to ketosis and ketoacidosis in critically ill adult patients with COVID-19 pneumonia and their correlation to ICU mortality.

Understandably, the prevalence of diabetes in patients with COVID-19 varies by age, region, and ethnicity [11]. Nearly 36% of COVID-19 patients had diabetes, according to an Italian study by Onder et al. (2020) [12]. A study from the United States by Bhatraju et al. (2020) reported that diabetes was associated with 58% of patients with COVID-19 [13]. We reported in our study that the prevalence of diabetes or prediabetes in ICU patients infected with the COVID-19 virus was 79.05% (200 patients out of 253 patients were diabetic or prediabetic). Wang et al. (2020) reported that out of 138 patients, 72% of COVID-19 patients with comorbidities including diabetes required admission to the ICU, compared to 37% of COVID-19 patients without comorbidities [14].

Diabetes and uncontrolled hyperglycemia have been reported as significant predictors of severity and mortality in patients infected with viruses, for example, the pandemic influenza A (H1N1) [15], MERS-CoV [16], and SARS-CoV [17]. Diabetes was considered an independent risk factor for complications and death during the 2002 to 2003 outbreak of severe acute respiratory syndrome (SARS-CoV-1) with mortality rates of 10% for SARS-CoV [1] and 36% in patients with MERS who had diabetes [18]. Similarly, the presence of diabetes tripled the risk of hospitalization and quadrupled the risk of ICU admission during Inﬂuenza A (H1N1) infection outbreak in 2009 [2].

Studies have also shown that COVID-19 is associated with hyperglycemia, particularly in elderly patients with type 2 diabetes [19]. Blood glucose control is important not only for patients who are infected with COVID-19 but also for those without the disease [20]. We noted in our study that peak blood glucose level was statistically significantly higher in nonsurvivors (mean 345 mg/dl) compared to survivors (mean 298 mg/dl) with a P-value of 0.006. However, HBA1C and blood glucose on admission showed no statistically significant change in mortality, which means that uncontrolled blood glucose during ICU admission plays a role in the final outcome for patients infected with COVID-19.

A correlation between the severity of the disease and diabetes was not clearly observed in some research conducted during the COVID-19 epidemic [21,22]. However, the evidence has remained controversial regarding whether diabetes itself increases susceptibility and influences outcomes from infections or whether the cardiovascular and renal comorbidities that are usually linked with diabetes are the key factors involved [8].

Diabetic patients with COVID-19 have been found to have a worse prognosis and higher mortality among hospitalized patients. A meta-analysis of nine studies from China (n = 1,936) showed a significant correlation between the severity of COVID-19 and diabetes (OR 2.67; 95% CI, 1.91-3.74; P < 0.01) [23]. However, a multivariate regression study revealed that the link between diabetes and mortality was no longer significant [20]. In the meta-analysis of nine trials (no 46,248) by Yang et al. (2020) [10], the odds ratio (OR) of severe COVID-19 was not significantly higher in diabetic patients (OR 2.07; 95% CI, 0.89-4.82).

Li et al. (2020), in a study of 658 hospitalized patients with confirmed COVID-19, reported that diabetes increased the length of hospital stay (median 33 vs. 17; P = 0.003) for patients with COVID-19 infection but had no effect on their mortality (33.3% vs. 14.8%; P = 0.313) [24]. Our study is similar to the study by Li et al. (2020) in that there was no significant difference regarding the mortality rate, (54.9% vs. 66%; P = 0.079) in diabetic patients versus non-diabetic patients admitted to the ICU in our study. Also, in our study, there was no significant difference regarding the length of ICU stay (median 15 vs. 16 days; P = 0.985) in diabetic patients versus non-diabetic patients because our study was concerned with ICU patients who were in critical illness regardless of their diabetic history, this explained why diabetes did not affect ICU mortality and length of ICU stay, while the study by Li et al. study was concerned with general hospitalized COVID-19 patients.

Our work found that there was no positive correlation between length of ICU stay and HBA1C (P =0.956), peak blood glucose level (P =0.582), and blood glucose on admission (P =0.838). Our results showed that diabetic history on admission and blood glucose on admission, will not affect the length of ICU stay and outcome but Peak blood glucose and other laboratory variables will correlate with mortality.

For more optimum results, we calculated the ROC curve to assess the relation between glycosuria, ketonuria, HBA1C, and blood glucose levels on ICU admission and mortality. We observed that there was no significant P value to predict ICU mortality, whereas peak blood glucose level has a prognostic value for ICU patients infected with COVID-19 (P value 0.004). The ROC curve in our study showed that uncontrolled hyperglycemia during ICU admission (peak blood glucose) and other laboratory variables (e.g., PCT (P = 0.047), and P < 0.001 of other laboratories e.g. Lactate, PH, WBC, D Dimer, Ferritin) are significant predictors of mortality in COVID-19 patients hospitalized in the ICU.

There are data regarding glucose metabolism in the body and the development of acute complications of diabetes, for example, diabetic ketoacidosis, in COVID-19 patients. Increased stress and production of hyperglycemic hormones, such as catecholamines and glucocorticoids, may be triggered by COVID-19 infection in diabetic patients, resulting in higher blood glucose levels and abnormal glucose variability [25].

Ketosis is defined by a positive test result of urine or serum ketone. Ketones are formed in the liver from free fatty acids [26]. When ketone consumption decreases, it results in ketosis (elevated blood ketone concentrations, e.g., acetone, acetoacetate, and β-hydroxybutyrate) [27].

Ketoacidosis, a severe metabolic illness marked by the accumulation of ketone bodies and acidosis, is most common in diabetics and rarely induced by other pathological conditions [28]. Ketoacidosis is defined by a positive test result of urine or serum ketone (above 0.6 mmol/L) and arterial PH < 7.35. DKA is a potentially fatal metabolic complication that is often caused by infections and linked to uncontrolled blood glucose. Seventy-six of the COVID-19 patients in our study were in ketosis, which suggests that COVID-19 may enhance the breakdown of fat and promote ketosis, leading to the development of ketoacidosis. DKA occurs as a result of insulin deficiency and increased counterregulatory responses that favor the production of ketones. It is possible that COVID-19 may aggravate pancreatic beta cell function and precipitate DKA [29].

We observed in our work that the ketosis group had a higher mortality rate (69.7% vs. 54.8%; P = 0.027) but no statistical difference in duration of ICU stay between ketosis and nonketosis groups (median 16 (1-115) vs. 14 (1-327) days; P < 0.335) and also between diabetic and nondiabetic groups (median 15 (1-197) vs. 16 (1-327) days; P < 0.985). In the current study, nine COVID-19 patients developed DKA; eight of them died. This suggests that COVID-19 infection could cause ketosis, or ketoacidosis, and induce DKA in those with diabetes. Ketosis increased ICU mortality but not the length of ICU stays.

Li et al. reported that 42 (6.4%) out of 658 patients presented with ketosis at hospital admission with COVID-19 [21]. Ketosis increased the mortality and length of hospital stay in their study. The ketosis group had a significantly higher mortality rate (21.4% vs. 8.9%; P = 0.017) like our study and also had a significantly longer hospital stay (median 19 (12.8-33.3) vs. 16 (10-24) days; P < 0.001). Our present study showed a longer ICU stay in the ketosis group admitted with COVID-19 (median 16 (1-115) vs. 14 (1-327) days; P < 0.335), but it was not significant due to different disease severity because most of our studied group were in critical illness with COVID-19 during ICU admission.

The study was limited because of the small number of patients with COVID-19 who progressed from ketosis to ketoacidosis. Notably, more research is needed to understand the mechanism of COVID-19-induced DKA. To lower the COVID-19-related mortality from complications, future research should focus on COVID-19 patients who have ketosis and ketoacidosis and monitor the disease’s long-term prognosis.

## Conclusions

We reported that COVID-19 infection can cause ketosis and induce DKA in diabetic people. There is a high prevalence of diabetes and ketosis among critically ill COVID-19 patients admitted to the ICU. Ketosis is associated with an increased mortality risk. Uncontrolled hyperglycemia is a significant predictor of mortality in critically ill COVID-19 patients. It is critical to control blood glucose levels in COVID-19 patients admitted to the ICU.
